# Health on the web: randomised trial of work-based online screening and brief intervention for hazardous and harmful drinking

**DOI:** 10.1186/1471-2458-13-505

**Published:** 2013-05-24

**Authors:** Elizabeth Murray, Zarnie Khadjesari, Stuart Linke, Rachael Hunter, Nick Freemantle

**Affiliations:** 1 Research Department of Primary Care and Population Health, University College London, Upper Floor 3, Royal Free Hospital, Rowland Hill Street, London, NW3 2PF, England; 2 Camden and Islington Mental Health and Social Care, Trust, Hill House (5th floor)17 Highgate Hill, London, N19 5NA, England

**Keywords:** Alcohol-related disorders, Alcohol, Screening and brief intervention, Internet, Randomised, Controlled trial, Workplace, Health promotion

## Abstract

**Background:**

Alcohol misuse is a significant international public health problem. Screening and brief intervention (SBI) in primary care reduces alcohol consumption by about 15 – 30%, sustained over 12 months in hazardous or harmful drinkers but implementation has proved difficult leading to growing interest in exploring the effectiveness of SBI in other settings, including the workplace. Computerised interventions for alcohol misuse can be as effective as traditional face-to-face interventions and may have advantages, including anonymity, convenience and availability.

**Methods/design:**

Individually randomised controlled trial to determine the effectiveness and cost-effectiveness of offering online screening and brief intervention for alcohol misuse in a workplace. Participants: adults (aged 18 or over) employed by participating employers scoring 5 or more on a three item screen for alcohol misuse (the AUDIT-C) indicating possible hazardous or harmful alcohol consumption, recruited through the offer of an online health check providing screening for a range of health behaviours with personalised feedback. Participants who accept the health check and score 5 or more on the alcohol screen will be randomised to receiving immediate feedback on their alcohol consumption and access to an online intervention offering support in reducing alcohol consumption (*Down Your Drink*) or delayed feedback and access to *Down Your Drink* after completion of follow-up data at three months. All employees who take the online health check will receive personalised feedback on other screened health behaviours including diet, physical activity, smoking, and body mass index. The primary outcome is alcohol consumption in the past week at three months; secondary outcomes are the AUDIT, EQ-5D, days off work, number and duration of hospital admissions, costs and use of the intervention. A sample size of 1,472 participants (736 in each arm) provides 90% power with 5% significance to determine a 20% reduction in alcohol consumption. Outcomes between groups at three months will be compared following the intention to treat principle and economic analyses will follow NICE guidance.

**Discussion:**

This innovative design avoids recruitment bias by not mentioning alcohol in the invitation and avoids reactivity of assessment by not collecting baseline data on alcohol consumption.

## Background

### Alcohol misuse is a public health problem

Alcohol misuse is a major international public health problem ranking third only to smoking and poor diet with physical inactivity as an avoidable cause of premature mortality and morbidity [1-3]. The World Health Organisation (WHO) defines three levels of alcohol misuse: hazardous drinkers (those drinking above recommended limits and hence at risk of harm, but not experiencing harm); harmful drinkers (drinking above recommended limits and experiencing harms); and dependent drinkers [4]. Most alcohol misusers are hazardous and harmful drinkers rather than dependent drinkers, for example, in England in 2004 it was estimated that approximately 26% of the population or some 8 million people misused alcohol, of whom over 7 million (21% of the population) were hazardous or harmful drinkers, compared to 1 million (3.6%) dependent drinkers [5].

Harms from excess alcohol consumption include physical and mental health problems for the individual and wider societal harms including domestic violence, marital breakdown, poor parenting, road traffic accidents, crime and loss of productivity at work [6,7]. The costs of alcohol misuse reflect this wide range of harms. The total cost of alcohol misuse in England was estimated at £20bn a year in 2004, made up of £1.7bn costs to the health service, £4.7bn costs of harm to family and society, £7.3bn costs of crime and antisocial behaviour and £6.4bn costs of loss of productivity at work – or some 17 million working days each year [6]. Similar figures have been reported from Australia, with up to 44% of workers drinking hazardously or harmfully [8] and some 7.4 million workdays lost at a cost of AUD 1.2bn [9].

### Evidence for effective interventions

There are effective interventions for hazardous and harmful drinkers. These include Screening and Brief Intervention (SBI) which consists of asking people how much they drink, and if this is above recommended guidelines, providing non-judgemental feedback and advice to cut down. SBI in primary care can result in a 15 – 30% reduction in alcohol consumption sustained for at least 12 months [10-12]. Despite the overwhelming evidence to support the widespread use of SBI in primary care there have been real problems with implementation, both in the UK and internationally [13]. Difficulties include reluctance on the part of patients to discuss their alcohol consumption with health professionals, reluctance on the part of health professionals to enquire about alcohol consumption for fear of damaging the doctor-patient relationship, lack of time in already complex consultations, widespread uncertainty about the recommended limits, and a shortage of appropriate services for non-dependent drinkers who request help with cutting down [14-17].

Alternatives to face-to-face SBI may include computerised interventions, with a recent systematic review finding some evidence that they are as effective in reducing alcohol consumption amongst adult hazardous and harmful drinkers as traditional face-to-face interventions [18]. On-line interventions have some advantages over face-to-face interventions for alcohol misuse, including convenience, easy access, anonymity (important in a stigmatised condition like alcohol misuse), and on-going availability (important in conditions with frequent relapses like alcohol misuse).

### Workplace screening and interventions for alcohol

Given the high prevalence and costs of alcohol misuse in the workplace it would seem sensible to explore the feasibility and effectiveness of delivering SBI at work [19]. However, there has been remarkably little work in this area. A systematic review of workplace interventions for alcohol published in 2009, identified 10 studies of which only 4 were randomised controlled trials and these all had methodological problems. However, within the limitations of the primary data, the authors concluded that brief interventions and interventions contained within health and lifestyle checks have potential to reduce alcohol consumption [20]. Studies looking specifically at web-based or computer-based programmes offering SBI in the workplace are rare, but include a pilot study by Matano et al. of the effects of an interactive web-based programme offered to Silicon Valley employees (n 145) which showed some benefit but the study was underpowered to show changes in alcohol consumption [21]. Doumas and Hannah compared web-based normative feedback with and without additional motivational interviewing with no intervention for young adults in the workplace (n 124) and found some benefit of the normative feedback but no additional benefit from the motivational interviewing component [22]. In Japan, Araki et al. compared face-to-face health education or e-mail health education to no intervention amongst 36 male workers at a manufacturing plant. Face to face health education had more impact than e-mail, with the latter appearing to have a small, non-significant impact on alcohol consumption [23]. Thus it seems timely to undertake a large randomised controlled trial of web-based screening and brief intervention in the workplace.

### Aims and objectives

The overall aim of the study is to determine the effectiveness and cost-effectiveness of offering on-line screening and brief intervention for alcohol misuse in a workplace. Specific objectives are to determine: the number of employees who complete the on-line screening questionnaire; the proportion of those who complete the on-line screening questionnaire whose score on the AUDIT-C suggests alcohol misuse; the proportion of those who score positive for alcohol misuse and are provided with feedback and access to the on-line intervention (*Down Your Drink*, DYD) who visit the intervention at least once; the effect on alcohol consumption of providing such feedback and access to DYD; and the costs and benefits of offering on-line SBI through the workplace.

## Design

**Figure 1:**
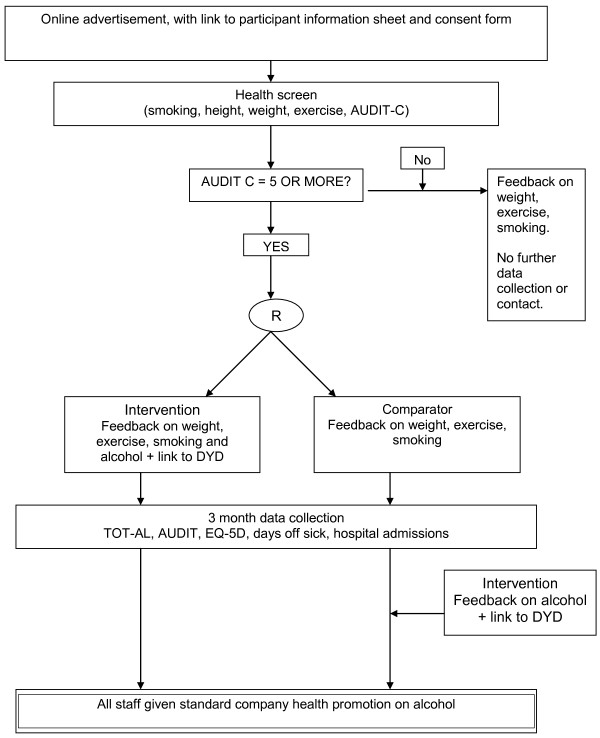
Health on the Web: Study flow-chart.

Individually randomised controlled trial (Figure 1).

### Ethics

Ethical approval has been obtained from UCL ethics committee (project ID number 3770/001).

### Setting

A large UK-based employer with an international workforce of approximately 100,000 has agreed to participate. This employer has a socially diverse workforce with a presence in the UK, Europe, North America, Australasia, Africa and the Middle East. The company has an active health promotion / occupational health programme with a regular series of health promotion campaigns and road shows. All employees have a work-related email address and most of the internal communication within the company is undertaken online.

### Target population

Adults with possible alcohol use disorders defined by scoring 5 or more on the AUDIT-C , an abbreviated version of the full AUDIT screening test for alcohol misuse developed by the WHO [24] and functioning e-mail addresses working for the participating employer.

### Inclusion and exclusion criteria

People who fulfill the following criteria will be eligible to participate in the trial:

Employees aged 18 or over with functioning e-mail addresses;

Provide informed consent;

Complete the online health check;

Score 5 or more on the AUDIT-C (three-item screening tool for alcohol misuse).

The only exclusion criterion is a score of 4 or less on the AUDIT-C.

### Recruitment

Employees will be offered the opportunity of an independent, confidential online health check with personalised feedback. The health check will be advertised on the home page of the company’s internal website, which all employees have to log on to daily. Interested staff will be asked to click on a hyperlink which will take them through to a participant information sheet and then an online consent form. Participants will be informed that the purpose of the study is to determine how best to use the web to improve health at work, that the study is being run by clinical and health service researchers at University College London, that participants will be asked to complete a questionnaire about their health-related behaviours which will form the basis for individualised feedback, and that a proportion of participants will also be asked for further follow-up data after three months. Participants will be assured that all data will be held confidentially in accordance with the Data Protection Act, that no-one from the company will know whether or not any individual has participated, and that individual data will not be shared with the employer, although the employer will be provided with anonymised aggregated data to inform future health campaigns.

### Intervention and comparator conditions

All employees who provide informed consent will be asked to complete the online health check. This health check will consist of a self-completion questionnaire assessing health behaviours and health state and will include baseline data. Thus the health check questionnaire will ask employees for demographic and occupational data as well as information on height, weight, smoking status, alcohol consumption, diet, level of physical activity, and health state.

All employees who complete the online health check will receive automated tailored feedback on their Body Mass Index (BMI), smoking, diet and level of physical activity. Data entered into the online health questionnaire are transferred automatically into a database and subjected to automated analysis which will allocate each response into a specific category. For example, respondents who enter height and weight data will have their BMI calculated automatically, and categorised as underweight, healthy weight, overweight, obese or morbidly obese. Feedback text will be written for each category. Responses for each health behaviour will be similarly categorised, so that the final feedback will be tailored for BMI, smoking status, diet and physical activity. Employees who score 4 or less on the AUDIT-C will be given feedback that they appear to be drinking at safe levels, with information on government recommended limits. These people will not be asked for further follow-up data and are not part of the trial.

Participants who score 5 or more on the AUDIT-C will be automatically entered into the trial and randomised to either the intervention or the comparator group. Participants randomised to the intervention group will receive the same individually tailored feedback on BMI, smoking status, diet and physical activity. In addition, they will be informed that the information they provided suggests that they are at increased risk of alcohol-related harm, and advised to reduce the amount they drink. The advice will acknowledge that it can be hard to cut down, and state that further support in achieving this is available from the online intervention, *Down Your Drink* (DYD), with an embedded hyperlink which participants can click on to be taken directly to DYD.

Participants who have been randomised to the comparator group will receive individually tailored feedback on BMI, smoking status, diet and physical activity. No feedback on alcohol consumption will be provided at this point. For ethical reasons, participants in the comparator group will be sent the feedback, advice and referral to DYD after completing the three month data collection procedures.

### Down Your Drink (DYD)

*Down Your Drink* is an on-line intervention for hazardous and harmful drinkers first developed by co-investigator Linke in 2000 with funding from the Alcohol Education and Research Council (AERC), updated in 2007 with support from the Medical Research Council (MRC) and National Prevention Research Initiative (NPRI) [25] and further updated for the purposes of this trial. The content is theoretically based, using motivational interviewing and cognitive behavioural techniques. The updated version used in this trial contains four sections called: *Should I cut down*?, *Planning to cut down*, *Cutting down*, and *Staying on track*. Interactive e-tools, such as the drinking episode diary provide opportunities for users to reflect on the role alcohol plays in their life and consider alternatives [25]. Within the programme there are warnings for users who are physically dependent on alcohol about the hazards of stopping drinking suddenly and the need to see their GP for help with detox.

### Outcomes

Outcomes have been selected to reflect the aims and objectives of the trial. Hence the primary outcome is past week alcohol consumption, measured by the TOT-AL [26]. This is an on-line measure of past week alcohol consumption that was developed for use in the online trial of *Down Your Drink* and has been validated in a UK population.

Secondary outcomes include:

The number of employees completing the online health check;

The number (proportion) of employees who complete the online health check and score 5 or more on the AUDIT-C;

The number (proportion) of participants in the intervention group who access DYD at least once;

Alcohol use disorders, measured by the AUDIT [4]. This will allow comparison of our data with other alcohol trials;

Outcomes for economic evaluation include:

Health state, measured by the EQ-5D [27];

Number of days of sickness absence in past 3 months (self-reported)[28];

Number and duration of hospital admissions in past 3 months (self-reported);

Costs, staff time and resources required to implement intervention

### Data collection

Baseline data collection will be integrated into the initial online health check questionnaire. This will include: year of birth, gender, occupational classification, height, weight, smoking status, dietary consumption of fruit and vegetables (portions per day), level of physical activity (minutes per week of vigorous, moderate and mild activity), frequency of consuming alcohol, average consumption on a drinking day, and frequency of drinking more than 6 drinks on one occasion (AUDIT-C), and health related quality of life (EQ-5D). Instruments for collecting data on hospital admissions will be those used in the SIPS and AESOPS trials of screening and brief intervention in multiple settings [29-31].

Follow-up data collection will be undertaken online. At follow-up participants will be asked to complete the TOT-AL, AUDIT, EQ-5D, report the number of days of sickness absence in the past 3 months and number and duration of any hospital admissions.

Data on numbers of health checks completed and proportion of employees scoring 5 or more on the AUDIT-C will be recorded. Use of DYD, including pages visited will be recorded using Google analytic software.

Data on costs of the intervention will be estimated from the costs incurred in the trial (separating out trial costs from intervention costs) for preparatory communications with stakeholders, preparing the invitations, developing the online health check, developing the tailored feedback, applying the software to provide tailored feedback, and updating.

Follow-up rates will be maximised by sending up to three e-mail requests for data. Non-responders will be contacted by post and phone to see if they received the e-mail reminders and request online completion. Participants who do not respond to these requests will be sent a final email and / or phoned asking just for primary outcome data (amount of alcohol consumed in past 7 days).

### Follow-up

The primary outcome is 3 months.

### Protection against bias

#### Selection bias

Randomisation will be performed centrally using automated randomisation software. Randomisation will be performed after collection of baseline data, and concealment of allocation will be complete, as there will be no way for either researchers or participants to know which arm a participant will be allocated to until after allocation.

#### Performance and detection bias (blinding)

Participants will not be aware that they are part of a trial. The consent information will indicate that the research focuses on how best to provide online health promotion at work, and will not mention alcohol specifically. As all data collection relies on either self-report or automated data collection researchers will be unable to affect responses at follow-up.

#### Attrition bias

Every effort will be made to maximise the response rates for follow-up data. Particular efforts will be made to ensure high response rates for the primary outcome.

#### Reporting bias

The trial protocol will be published. Analyses will follow the intention to treat principle and will be pre-specified.

### Sample size

736 participants in each arm at 3 month follow up gives us 90% power with 5% significance to determine a 20% reduction in alcohol consumption. Allowing for up to 25% loss to follow-up at 3 months we will need to randomise 920 participants to each group (n 1,840); however we will take extensive steps to minimise loss to follow-up as described above.

### Analyses

Analyses will compare outcomes between groups at three months following the intention to treat principle. The principal analysis will be conducted using a generalised linear model with identity link and Gaussian error. The baseline AUDIT-C score will be included as a patient level explanatory variable, along with a classification variable for workplace and for randomised group. Analogous models will be conducted for secondary outcomes. We will conduct an additional prognostic model examining subject and organisational factors (and potential interactions) associated with the size of any treatment effect. For the economic evaluation we will design a decision analytical model in line with National Institute of Health and Clinical Excellence (NICE) guidance to determine the incremental cost per quality adjusted life year (QALY) gained, calculated using the EQ-5D, and cost per reduction in units of alcohol consumed of the web-based screening and brief intervention compared to web-based screening alone at 3 months, 12 months and 10 years. As participants in the control group will be referred to DYD at 3 months, the 12 month analysis for the control group will assume that the costs and outcomes for the first 3 months of the trial would have continued for the next 9 months had they not received the intervention at 3 months. Effectively, costs and outcomes over 12 months for the control group will be cost and outcomes for the first 3 months multiplied by 4. Published data will be used for the 10 year model and all costs and outcomes will be discounted in accordance with NICE guidance. The primary cost- effectiveness analysis will be from the perspective of the NHS with a secondary cost-offset analysis from the perspective of the employer. Costs to the NHS will include the costs of hospital admissions. Unit costs will be taken from standard published sources such as Personal Social Services Research Unit (PSSRU) [32]. The cost to the employer will include the cost of implementing web based screening and brief intervention and the cost of the time it takes employees to complete web based screening and brief intervention. This will be offset by any cost savings associated with reduced number of days of sick leave for the screening and brief intervention group compared to the screening only group. We are aware of the potential seasonal variation in sickness absence and will address this in the sensitivity analyses. Analyses will be subject to deterministic and probabilistic sensitivity analyses. Modelling will be used to explore the relative cost-effectiveness of primary care versus work-place SBI.

## Discussion

The design of the trial raises some interesting methodological and ethical issues, discussed here.

### Comparator and duration of follow-up

The trial uses a wait list design, with the comparator group receiving no feedback on their alcohol consumption until they have completed data collection at the primary outcome point (3 months). We consider this is ethical as a) this is a non-help seeking population; b) the time-course of alcohol misuse is long (many years) and it is unlikely that three months delay in provision of feedback and advice is likely materially to alter outcomes; and c) all participants are advised to consult their GP if they have any concerns about their health. We had initially considered a 12 month primary outcome point but decided this was potentially unethical as 12 months delay could potentially be harmful. Moreover, our procedures had to be acceptable to the collaborating workplace, which had indicated that a three month follow-up period was the longest they would consider acceptable. Our previous work had suggested that reductions in alcohol consumption seen at 3 months were maintained at 12 months [33], although the data from that trial may not apply to the different population recruited here.

### Dependent drinkers

A very small proportion of participants are likely to be dependent drinkers. NHS Information Centre statistics for 2007 report the prevalence of moderate or severe dependence as 1% in men and 0% in women in the general adult population. An additional 8.3% of men and 3.6% of women have mild dependence. People with moderate or severe dependency require detoxification under medical supervision, but those with mild dependence do not. We are not assessing level of dependency, and will not be able to identify participants who are moderately or severely dependent on alcohol. Down Your Drink provides information to users about dependency, including warnings not to stop consumption suddenly and advice to seek medical help.

### Baseline data collection

Collecting baseline data on alcohol consumption would increase the power of the study, as it would allow for determination of individual change. However, alcohol consumption is notoriously sensitive to assessment, with simple measurement of past week alcohol consumption or completion of the Alcohol Use Disorders Identification Test (AUDIT) resulting in a decrease in consumption of about 20% [34]. This reactivity of assessment is thought to be a contributing factor to the lack of difference seen in many alcohol trials [35] and for this reason, we decided against collecting baseline data on alcohol consumption.

### Advantages and disadvantages of online trials

Advantages of conducting trials on-line compared to conventional trial methods include rapid and easy recruitment, automated randomisation with concealment of allocation, and automated data collection [36]. For example, our on-line trial of DYD recruited nearly 8,000 participants over 2 years with no advertising and no marginal cost per additional participant recruited [33]. Automated data collection, whereby participants are automatically sent e-mails requesting follow-up data with embedded hyperlinks in the e-mail, allowing participants to access questionnaires with one click, is relatively cheap, and because participants enter data online, it can be automatically transferred to the trial database, obviating the need for manual data entry and preventing transcription errors. Thus large trials can be undertaken at relatively low cost. There are disadvantages to conducting trials on-line, including difficulty in describing the population from which the sample was drawn and knowing what proportion of potential participants actually participated (affecting external validity), and low follow-up rates (affecting internal validity). Low follow-up rates are probably related to the relatively impersonal nature of on-line trials and the ease with which e-mail reminders can be ignored or deleted. This proposal capitalises on the advantages of on-line trials while avoiding the disadvantages. Recruiting from a work-force ensures we know the total number of employees contacted, and also allows us to use multiple forms of follow-up, including telephone and post, thus improving retention.

## Abbreviations

AUDIT: Alcohol use disorders identification test; AUDIT-C: Three item screening test derived from the AUDIT; BMI: Body mass index; DYD: Down your drink; NICE: National Institute of Health and Clinical Excellence; NHS: National health service; PSSRU: Personal social services research unit; QALY: Quality adjusted life year; SBI: Screening and brief intervention; TOT-AL: Total past week alcohol consumption – an online measure of past week alcohol consumption; UK: United Kingdom; WHO: World health organisation.

## Competing interests

The authors declare they have no competing interests

## Authors’ contributions

EM and ZK conceived and designed the study with contributions from all authors. SL designed the original DYD and with ZK modified it for use in this setting. NF designed the statistical analyses and RH the health economic analyses. All authors read and approved the final manuscript.

## Pre-publication history

The pre-publication history for this paper can be accessed here:

http://www.biomedcentral.com/1471-2458/13/505/prepub
